# Eyes Wide Shopped: Shopping Situations Trigger Arousal in Impulsive Buyers

**DOI:** 10.1371/journal.pone.0114593

**Published:** 2014-12-09

**Authors:** Benjamin G. Serfas, Oliver B. Büttner, Arnd Florack

**Affiliations:** 1 Department of Applied Psychology: Work, Education, and Economy, University of Vienna, Vienna, Austria; 2 Department of Economic Sciences, Zeppelin University, Friedrichshafen, Germany; University Hospital of Bellvitge-IDIBELL; CIBER Fisiopatología Obesidad y Nutrición (CIBERObn), Instituto Salud Carlos III; Department of Clinical Sciences, School of Medicine, University of Barcelona, Spain, Spain

## Abstract

The present study proposes arousal as an important mechanism driving buying impulsiveness. We examined the effect of buying impulsiveness on arousal in non-shopping and shopping contexts. In an eye-tracking experiment, we measured pupil dilation while participants viewed and rated pictures of shopping scenes and non-shopping scenes. The results demonstrated that buying impulsiveness is closely associated with arousal as response to viewing pictures of shopping scenes. This pertained for hedonic shopping situations as well as for utilitarian shopping situations. Importantly, the effect did not emerge for non-shopping scenes. Furthermore, we demonstrated that arousal of impulsive buyers is independent from cognitive evaluation of scenes in the pictures.

## Introduction

Buying impulsiveness is consumers' tendency to engage repeatedly in spontaneous, “on-the-spot” purchases, without consideration of potential consequences [1]. Buying impulsiveness has been conceptualized as a one-dimensional construct that reflects individual differences in purchasing behavior along a continuum ranging from non-impulsive buyers to highly impulsive buyers [1] [2]. Typical characteristics of impulsive buyers are that they neglect the consequences of their purchasing behavior, and might experience emotional conflicts [3].

Research on impulsive buying commonly uses questionnaires or self-ratings. Thus, it often neglects consumers' physiological reactions, though these reactions might represent an important link between individual traits and behavior [4]. For instance, hormone balance influences spending behavior [5]. The physiological reaction we suggest to be crucial in impulsive buying is arousal.

An expression used to indicate a state of arousal, as well as that of excitement and joy, is “wide-eyed.” However, “wide-eyed” may be more than just a phrase: Previous research suggests that our eyes literally widen in situations in which individuals experience arousal, as demonstrated by physiological literature using pupil responses. Task-evoked pupil dilation is a common measure in psychophysiology [6]. Previous research has shown that pupil dilation is linked to affective arousal [7]. For instance, pupils dilate due to pleasant sounds [8] or valuable cues [9]. However, research on impulsive buyers' physiological reaction is scarce. A notable exception is a recent fMRI study, which demonstrated that buying impulsiveness accounts for differences in neural activation patterns due to perceptions of attractive brands [10]. Additionally, impulsive buyers seem to be particularly vulnerable to tempting stimuli in shopping situations [11]. Thus, a high level of buying impulsiveness might be accompanied by physiological responses, specifically arousal.

There might be two reasons why shopping situations trigger arousal in impulsive buyers: self-control conflicts and affective responses. Most theories agree that failures in self-control play an important role in buying impulsiveness [12] [13] [14]. A central point of self-control is to regulate inner-subject processes, such as desires and impulses [15]. Struggling with self-control conflicts elicits arousal [16]; consequently, buying impulsiveness can be assumed to be accompanied by physiological arousal.

Impulsive buying is related not only to a lack of self-control, but also to increased affective responses [10]. Indeed, Babin, Darden, and Griffin [17] found buying impulsiveness to be linked to hedonic shopping; that is, shopping for mere enjoyment. Consumers' joy is closely linked to arousal, and this arousal at the point of purchase is an important factor to explain shopping behavior [18]. However, research on buying impulsiveness with psychophysiological measures of arousal is currently lacking.

Furthermore, we assume that arousal in impulsive buyers is domain-specific. Recent research has demonstrated that temptations in self-control conflicts are largely idiosyncratic and domain-specific [19]. Results of a recent study demonstrated that impulsive buyers are more likely to be visually distracted by goal-irrelevant products, but only when the task is framed as a shopping situation [11]. Similarly, we assume a domain-specific arousal in impulsive buyers. Specifically, we assume that buying impulsiveness is correlated with intensity of arousal in shopping scenes, but not in non-shopping scenes.

The present study is the first to use a physiological measure to examine processes that underlie buying impulsiveness. Specifically, we suggest arousal to be a crucial mechanism underlying buying impulsiveness. In order to address the role of arousal in shopping situations, we measured pupil dilation while participants viewed pictures of non-shopping and shopping scenes. To demonstrate the effect across a variety of shopping situations, we used pictures of shopping scenes that are typically considered hedonic, such as apparel shopping, and shopping scenes that are typically considered as utilitarian, such as grocery shopping [20]. We expected that arousal in response to shopping scenes (as reflected in pupil dilation) is closely associated with the level of buying impulsiveness. As we expect a domain-specific response, this is only true for shopping scenes. In contrast, for non-shopping scenes, highly impulsive and low-impulsive buyers should not differ regarding arousal. Additionally, we propose that arousal in impulsive buyers is direct physiological reaction, which is independent from the cognitive evaluation of scenes in the pictures.

## Method

### Ethics statement

According to the Austrian Universities Act 2002 (UG2002), which was in place at the time the study was carried out, only medical universities were required to appoint ethics committees for clinical tests, application of medical methods, and applied medical research. Consequently, no ethical approval for this specific study was required.

Nevertheless, the present study was part of a project that was funded by a European Union Marie Curie FP7 Integration Grant, and the project received ethical approval. As there was no institutional review board in charge at the time the project proposal was submitted, it was approved by an independent expert (Institute for Ethics and Law in Medicine, University of Vienna).

The present study was conducted in accordance with the Declaration of Helsinki (revised 1983) and local guidelines of the Faculty of Psychology, University of Vienna. Written informed consent was given by all participants, who could also withdraw at any time during the experiment without further consequences. At the end of the experiment, participants were debriefed in detail.

### Participants

Fifty participants completed the study, and each received six euros as compensation. We recruited participants, without exclusion criteria, from the local subject pool. The subject pool consists of volunteers who are recruited online, as well as via flyers and posters, at the university. As studies that are conducted in our lab regularly involve paradigms from psychological research, psychology students are not included in the subject pool, in order to avoid demand effects that result from prior knowledge of these paradigms.

Participants were unaware that physiological reaction to picture content was the focus of the study, and they were not familiar with the stimulus material. We removed one participant due to missing values in the rating task, resulting in a sample of 49 participants (*M*_age_ = 25.4, *SD*_age_ = 5.3, 31 women).

### Material

We used 40 different pictures that belonged to one of four categories: (a) hedonic shopping context, (b) utilitarian shopping context, (c) positive non-shopping context, and (d) neutral non-shopping context. Pictures in the shopping context depicted situations in various stores. For instance, the hedonic shopping context included pictures of the interior of apparel stores and of shopping bags with clothing. The utilitarian shopping context, on the other hand, included pictures of the interior of grocery stores and carts containing groceries. As stimuli for the non-shopping situations, we used a subset of pictures used in a previous study measuring pupil dilation [4]. The subset consisted of pleasant and neutral pictures from the International Affective Picture System [21].

In order to rule out the possibility that visual aspects influenced pupil dilatation, the pictures were transformed to 8-bit greyscale. Each picture had the same mean luminosity (RGB: 127,127,127).

A pretest ensured adequate picture selection, and confirmed the comparability of visual aspects: there were no differences regarding perceived complexity and contrast between the four categories of pictures. Additionally, participants in the pretest rated positive pictures as more pleasant compared to pictures in all other categories, and shopping pictures as more strongly related to shopping compared to pictures of non-shopping scenes. The sample used in the pretest was comparable regarding age (*M*_age_ = 24.3, *SD*_age_ = 4.1) with the sample of the main study (*M*_age_ = 25.4, *SD*_age_ = 5.3). The proportion of women in the pretest (86%) was higher than that of the main study (63%), but as the pretest did not find any gender effects regarding picture rating this difference is negligible.

### Procedure and measures

The study was conducted in individual sessions. Participants were verbally informed that the study was about visual perception. Further instructions were given on a computer screen. The instruction stated that the participants would see several pictures, and that their task would be to rate the pictures. The instructions also explained the process of the experiment to the participants: that (a) they would see a cross in the middle of the screen, (b) the cross disappears when they look at it, (c) the picture is presented for a few seconds, and (d) they would be asked for their rating.

The presentation screen was a 22-inch monitor with a resolution of 1680×1050 pixels and a refresh rate of 60 Hz. Participants were seated approximately 60 cm away from the monitor. Lighting was kept constant and the participants wore headphones to control for background noises. The grey background during the task was equal to the mean luminosity of the pictures (RGB: 127,127,127).

The experiment consisted of 40 trials. Each trial started with a fixation cross in the location where the pictures were later presented. After participants had fixated on the cross for 1,500 ms, a blank screen was presented for 500 ms. Then, one of the 40 pictures from the four categories (neutral and positive non-shopping situations, hedonic and utilitarian shopping situations) was presented on the screen for 4,000 ms. The order of the pictures was fully randomized. During the picture presentation, we assessed pupil diameter using an SMI RED 500 remote eye-tracker (Sensomotoric Instruments GmbH, Teltow, Germany) with a sampling rate of 250 Hz. After the presentation of each picture, the participants rated how much they liked the picture (1 =  not at all, 7 =  very much) and how positively they perceived the scene presented in the picture (i.e., valence; 1 =  negative, 7 =  positive).

After the 40 trials, the participants answered a questionnaire that contained questions on demographics and the *buying impulsiveness scale* (BIS;α = .83) from Rook and Fisher [1]. The BIS consists of nine items, such as “I often buy things spontaneously.” Participants indicated on a five-point rating scale how much they agreed with each statement (1 =  strongly disagree, 5 =  strongly agree). The authors of the BIS propose that buying impulsiveness represents a continuum; thus, the BIS does not have a cut-off point, but uses the scale score as a continuous measure of buying impulsiveness (possible range: 9–45, observed range: 9–36, *M* = 18.3, *SD* = 6.1).

### Data on pupil dilation

We corrected for artifacts and calculated tasked-evoked pupil dilation according to standard procedures [6]. Specifically, we excluded blinks and corrected for unrelated events (such as movements and accommodation responses). Pupil diameter during the fixation-cross period prior to each picture functioned as baseline. We calculated task-evoked pupil dilation, as response to the different scenes in the picture, by subtracting the baseline from the average pupil diameter during the picture presentation. This tasked-evoked pupil dilation served as our measure of arousal [6].

## Results

### Physiological arousal

We hypothesized that context (non-shopping vs. shopping) moderates the influence of buying impulsiveness on arousal. We conducted a general linear model analysis with pupil dilation as the dependent variable; the context was a within-subject factor, and buying impulsiveness was a continuous between-subject predictor.

**Figure 1:**
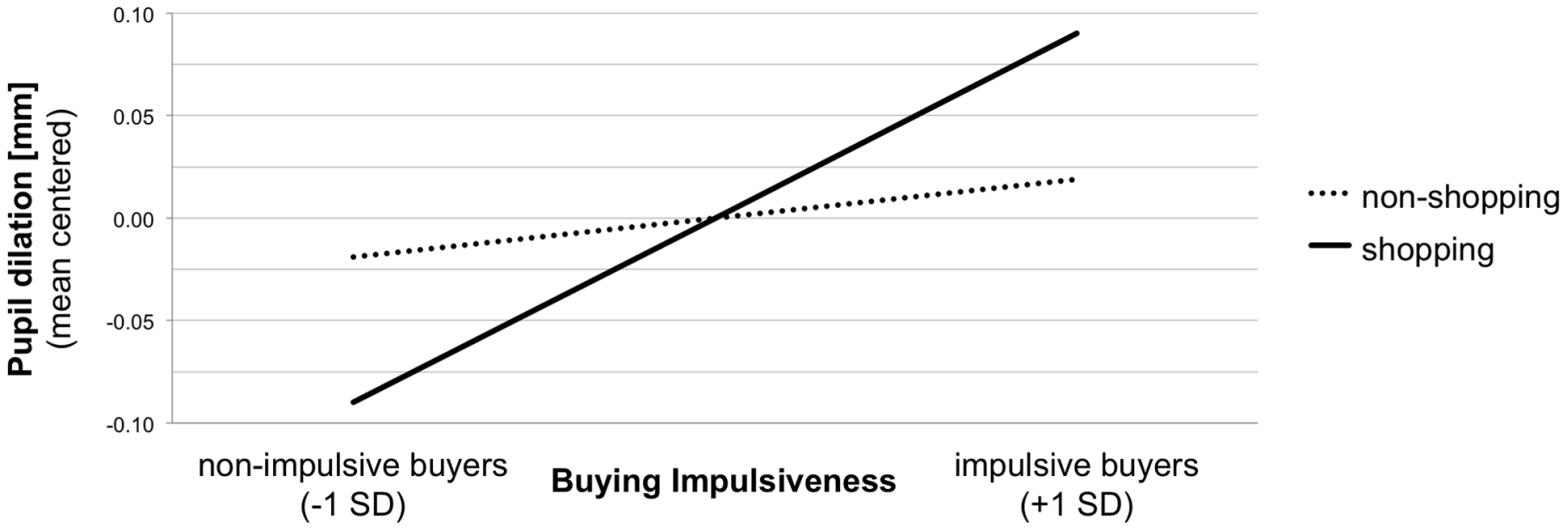
Simple slope regression lines for pupil dilation predicted by buying impulsiveness and context.

The effect of context was significant, *F*(1, 47)  = 16.18, *p*<.001, whereas the effect of buying impulsiveness was not, *F*(1, 47) = 2.58, *p* = .12. The respondents' pupils became more dilated while viewing shopping scenes compared to non-shopping scenes. In line with our expectation, a significant Context x Buying Impulsiveness interaction, *F*(1, 47) = 5.81, *p* = .02, indicated a differential influence of buying impulsiveness in each context (Fig. 1).

We analyzed the nature of the interaction by regressing pupil dilation on the continuous measure of buying impulsiveness in each context. Buying impulsiveness significantly predicted pupil dilation in the shopping context, but not in the non-shopping context (Table 1). When viewing pictures of shopping scenes, pupil dilation increased as a function of buying impulsiveness. Hence, a high level of buying impulsiveness was accompanied by stronger pupil dilation compared to a low level of buying impulsiveness. This applied to the hedonic as well as to the utilitarian shopping pictures (Table 1), although the effect was only marginally significant for utilitarian shopping pictures.

**Table 1:** *Regression coefficients split by context.*

	*B*	*SE*	*β*	*t*	*df*	*p*
Shopping	0.09	0.04	.34	2.49	47	.016
Hedonic	0.11	0.04	.37	2.72	47	.009
Utilitarian	0.06	0.04	.25	1.78	47	.08
Non-shopping	0.02	0.04	.08	0.52	47	.61
Neutral	0.03	0.04	.09	0.61	47	.55
Positive	0.01	0.04	.05	0.31	47	.76

*Note.* Dependent variable: pupil dilation [mm]; independent variable: buying impulsiveness [z-standardized].

### Picture rating

In order to examine whether the arousal was independent from cognitive evaluation of the shown scenes, we analyzed whether buying impulsiveness influenced the picture ratings. We conducted two general linear model analyses, with liking of the pictures and valence of the scenes depicted in the pictures as dependent variables, respectively. Context was a within-subject factor and buying impulsiveness was a continuous between-subject predictor. Overall, the participants liked the non-shopping pictures more, *F*(1, 47) = 67.42, *p*<.001, and rated scenes in non-shopping pictures as more positive, *F*(1, 47) = 93.00, *p*<.001. However, neither liking, *F*(1, 47) = 0.02, *p* = .90, nor valence, *F*(1, 47) =  0.13, *p* = .72, were influenced by buying impulsiveness. In addition, the analyses revealed no effects of the Context x Buying Impulsiveness interaction neither for liking, *F*(1, 47) = 0.32, *p* = .57, nor for valence, *F*(1, 47) = 1.32, *p* = .26. Additionally, pupil dilation did not correlate with liking or valence, neither in the shopping context, *r*(47)s<−.10, *p*s>.50, nor the non-shopping context, *r*(47)s<.14, *p*s>.33. Taken together, these findings indicate that buying impulsiveness did not influence physiological arousal through cognitive evaluations of the scenes in the pictures.

## Discussion

The present study is the first to analyze the effect of buying impulsiveness on arousal using a physiological measure. While participants viewed and rated pictures of shopping and non-shopping scenes we measured pupil dilation, representing arousal. For pictures depicting shopping scenes, high buying impulsiveness was accompanied by an increased intensity of arousal, as reflected in the participants' pupil dilation. Importantly, for non-shopping scenes the intensity of arousal was not correlated with buying impulsiveness. This specificity of reaction is in line with research indicating that impulsivity is idiosyncratic and domain-specific [11] [19].

Previous research has shown that the act of purchasing is linked to arousal [18]. The present research is the first to demonstrate that shopping situations without any act of purchasing—even without any possibility to purchase—already trigger arousal in impulsive buyers. When confronted with pictures of shopping scenes, consumers with a high level of buying impulsiveness became more aroused compared to consumers with a low level of buying impulsiveness. Importantly, this did not hold for non-shopping scenes. These results suggest that the physiological reaction of impulsive buyers does not depend on an act of purchasing. A shopping situation alone seems to be sufficient to trigger arousal in impulsive buyers.

The finding that shopping situations, in and of themselves, trigger arousal is substantiated by the fact that the type of the shopping scene did not have an effect. Arousal of highly impulsive buyers was not restricted to specific shopping scenes; but buying impulsiveness is linked to intensity of arousal in a wide range of shopping scenes, e.g. grocery shopping or apparel shopping. This implies that physiological arousal triggered by shopping situations is independent from the type of shopping situation.

Furthermore, we found that the self-reported evaluation of the shopping scenes was independent from buying impulsiveness. This suggests that the correlation between buying impulsiveness and arousal is independent from cognitive evaluations. We assessed how much participants liked the pictures, and whether the scene in the pictures was positive or negative. Impulsive buyers rated shopping scenes equally to non-impulsive buyers. Thus, pupil dilation was not related to evaluation of the scenes depicted in the pictures. Hence, evaluation cannot be a mediating factor for the moderated effect of buying impulsiveness and context on arousal. This fact further stresses our argument that shopping situations trigger arousal in and of themselves.

Based on the physiological reactions of impulsive buyers on pictures of shopping scenes, we conclude that impulsive buyers feel aroused just by entering a shopping environment and that the act of purchasing is not necessary to stimulate an aroused state in impulsive buyers. Moreover, the results indicate that this arousal of impulsive buyers is a basic physiological reaction that is independent from cognitive evaluations of the shopping scene.
